# Identification of Mechanisms by Which Genetic Susceptibility Loci Influence Systemic Sclerosis Risk Using Functional Genomics in Primary T Cells and Monocytes

**DOI:** 10.1002/art.42396

**Published:** 2023-04-09

**Authors:** David González‐Serna, Chenfu Shi, Martin Kerick, Jenny Hankinson, James Ding, Amanda McGovern, Mauro Tutino, Gonzalo Villanueva‐Martin, Norberto Ortego‐Centeno, José Luis Callejas, Javier Martin, Gisela Orozco

**Affiliations:** ^1^ Institute of Parasitology and Biomedicine López‐Neyra, Consejo Superior de Investigaciones Científicas (IPBLN‐CSIC) Granada Spain; ^2^ Division of Musculoskeletal and Dermatological Sciences, Centre for Genetics and Genomics Versus Arthritis, School of Biological Sciences, Faculty of Biology, Medicine and Health The University of Manchester Manchester UK; ^3^ Division of Infection, Immunity and Respiratory Medicine, School of Biological Sciences, Faculty of Biology, Medicine and Health The University of Manchester Manchester UK; ^4^ Department of Internal Medicine, Hospital Universitario San Cecilio Institute for Biosanitary Research of Granada (ibs.GRANADA) Granada Spain; ^5^ Division of Musculoskeletal and Dermatological Sciences, Centre for Genetics and Genomics Versus Arthritis, School of Biological Sciences, Faculty of Biology, Medicine and Health, The University of Manchester, and NIHR Manchester Biomedical Research Centre Manchester University NHS Foundation Trust, Manchester Academic Health Science Centre Manchester UK

## Abstract

**Objective:**

Systemic sclerosis (SSc) is a complex autoimmune disease with a strong genetic component. However, most of the genes associated with the disease are still unknown because associated variants affect mostly noncoding intergenic elements of the genome. We used functional genomics to translate the genetic findings into a better understanding of the disease.

**Methods:**

Promoter capture Hi‐C and RNA‐sequencing experiments were performed in CD4+ T cells and CD14+ monocytes from 10 SSc patients and 5 healthy controls to link SSc‐associated variants with their target genes, followed by differential expression and differential interaction analyses between cell types.

**Results:**

We linked SSc‐associated loci to 39 new potential target genes and confirmed 7 previously known SSc‐associated genes. We highlight novel causal genes, such as *CXCR5,* as the most probable candidate gene for the *DDX6* locus. Some previously known SSc‐associated genes, such as *IRF8, STAT4*, and *CD247*, showed cell type–specific interactions. We also identified 15 potential drug targets already in use in other similar immune‐mediated diseases that could be repurposed for SSc treatment. Furthermore, we observed that interactions were directly correlated with the expression of important genes implicated in cell type–specific pathways and found evidence that chromatin conformation is associated with genotype.

**Conclusion:**

Our study revealed potential causal genes for SSc‐associated loci, some of them acting in a cell type–specific manner, suggesting novel biologic mechanisms that might mediate SSc pathogenesis.

## INTRODUCTION

Systemic sclerosis (SSc) is a complex, chronic, immune‐mediated disease that affects the connective tissue, characterized by an immune imbalance, vascular alterations, and an excessive collagen deposition leading to fibrosis (1). Several lines of evidence implicate T cells and monocytes/macrophages as important cell types in SSc pathogenesis (2, 3), and modifications in the proportions of CD4+ T cell subpopulations and their functional alterations may contribute to the vascular dysregulation and fibrosis observed in the disease (4, 5). In addition, circulating monocytes/macrophages with a profibrotic phenotype are increased in blood from SSc patients (6, 7), and changes in human monocyte‐derived macrophage transcriptome are related to SSc genetic variants (8).

SSc has a complex genetic component, and its etiology is poorly understood. Large‐scale genetic studies have so far identified 27 independent signals associated with susceptibility to SSc (9, 10). Interestingly, many of the different genes assigned to SSc‐associated loci determined by genome‐wide association studies (GWASs) are related to T cell activation and macrophage regulation pathways (11, 12), and are shared among immune‐mediated diseases. This may be of interest in drug repositioning for rare diseases like SSc, for which there are no specific available treatments (13). However, the majority of single‐nucleotide polymorphisms (SNPs) associated with SSc map to noncoding regions of the genome that are enriched in enhancer elements, which are often specific to cell type (14, 15). These regulatory elements can interact with genes often located hundreds of kilobases away, bypassing nearby genes in many cases (16).

Thus, the current challenge remains in linking disease‐associated regions with the genes that they affect and in the specific cell types involved to pinpoint the mechanisms and the biologic pathways implicated in genetically susceptible patients (17). Many techniques to analyze the 3‐dimensional genome architecture have emerged, such as chromosome conformation capture (18, 19), that can help annotate these genetic variants. The most powerful technique developed to date, Hi‐C, allows the identification of chromosomal interactions genome wide (20). A more recent technique, capture Hi‐C, allows enrichment of specific chromosomal regions of interest, such as disease risk loci (region capture Hi‐C) or promoters (promoter capture Hi‐C) from Hi‐C libraries in a cost‐effective way (21). This technique has been successfully applied in different cell types to link enhancers and noncoding disease variants to potential target genes (22). Previously, we successfully applied capture Hi‐C using cell lines to identify disease‐causal genes and potential drugs for repositioning in autoimmune diseases (23, 24). Since the regulation of gene expression and chromatin conformation is highly context specific, it is essential to apply these technologies to primary cells isolated from patients. Moreover, recent evidence points to alterations in chromatin conformation linked to genotype, but these studies have so far only been carried out in a limited way and in cell lines (25, 26).

In this study, we used promoter capture Hi‐C in 2 of the most relevant cell types in SSc pathogenesis, CD4+ T cells and CD14+ monocytes, from SSc patients and healthy controls to annotate gene targets within all known SSc‐associated GWAS loci. We also integrated these data with RNA sequencing, expression quantitative trait loci (QTLs), and genotype information to create a multiomic approach to identify interactome and transcriptome differences between cell types and disease state that could be of interest in the pathogenesis of SSc.

## PATIENTS AND METHODS

### Isolation of CD4+ T cells and CD14+ monocytes

Primary CD4+ T cells and CD14+ monocytes were collected from 10 SSc patients and 5 healthy individuals. All SSc patients were diagnosed according to the American College of Rheumatology/EULAR 2013 criteria for SSc (27). Cohort characteristics are described in Supplementary Table 1, available on the *Arthritis & Rheumatology* website at https://onlinelibrary.wiley.com/doi/10.1002/art.42396. All patients and healthy controls gave written informed consent, and the study was approved by the local ethics committees.

Peripheral blood mononuclear cells (PBMCs) were isolated from 70‐ml blood samples using Ficoll density‐gradient centrifugation. An EasySep Human CD14+ Selection kit (product no. 17858; StemCell Technologies) was used to isolate CD14+ cells from PBMCs and, subsequently, an EasySep CD4+ T Cell Isolation kit (product no. 17952; StemCell Technologies) was used to isolate CD4+ T cells from the remaining PBMCs, according to the manufacturer's instructions.

### Capture Hi‐C library generation and processing

Five to 10 million isolated CD4+ T cells and CD14+ monocytes were crosslinked in 1% formaldehyde; the reaction was then quenched with 0.125 *M* glycine. Each Hi‐C library was prepared from fixed cells using an Arima HiC kit (Arima Genomics) and a KAPA HyperPrep kit (Roche), following the manufacturers’ protocols. Hi‐C samples were then hybridized with the SureSelect custom capture library using Agilent SureSelect XT HS reagents (product nos. G9702A and G9496A), following the manufacturer's protocols.

Reads were mapped on the GRCh38 genome with HiCUP software version 0.7.4 (28) and Bowtie2 software version 2.3.2 (statistical data provided in Supplementary Table 2 at https://onlinelibrary.wiley.com/doi/10.1002/art.42396). Significant chromatin interactions were identified using CHiCAGO software version 1.13.1 (29) and a threshold CHiCAGO score >5 in different conditions for cell type (CD4+ T cells from 15 samples and CD14+ monocytes from 15 samples), and cell type and disease state (CD4+ T cells from 10 SSc patients and 5 healthy controls and CD14+ monocytes from 10 SSc patients and 5 healthy controls). Principal components analysis was performed in each cell type in order to detect potential biases (Supplementary Figure 1, https://onlinelibrary.wiley.com/doi/10.1002/art.42396).

Chicdiff software version 0.6 (30) was used to detect differential interactions between different conditions as follows: CD4+ T cells versus CD14+ monocytes, CD4+ T cells from SSc patients versus CD4+ T cells from healthy controls, and CD14+ monocytes from SSc patients versus CD14+ monocytes from healthy controls. For each comparison, only those interactions with a CHiCAGO score >5 in at least one condition were included in differential analysis. Differential interactions with a weighted adjusted *P* value less than 0.05 were identified as significant. Spearman's rank correlation was performed to test the correlation of log_2_ fold change values in differential interactions between patients and controls in CD4+ T cells and CD14+ monocytes.

### Genotype calling and allele‐specific analysis

Genotypes were called using Glimpse software version 1.1.1 (31) from capture Hi‐C reads aligned using HiCUP (28) to a masked GRCh38 genome. Genotype phasing was then carried out using the integrated phasing pipeline (32), which integrates population phasing with reads phasing using the capture Hi‐C reads. Reads were then split using SNPsplit software version 0.5.0 (33), generating allele‐specific alignments. Counts for each allele and for each CHiCAGO significant loop were then calculated using BEDTools software version 2.30 (34), and data were integrated in Python 3.9. Allelic imbalance of the reads was tested using a binomial test for each sample that was heterozygous for that SNP and satisfied some requirements (see Supplementary Methods, https://onlinelibrary.wiley.com/doi/10.1002/art.42396). All the resulting *P* values were then checked for directionality and merged using the Fisher's method for meta‐analysis. Resulting *P* values were then corrected using the Benjamini‐Hochberg method.

### RNA‐sequencing library generation and processing

RNA was isolated from 500,000 purified cells using the RNeasy Micro kit (product no. 74004; Qiagen). Libraries were generated using Illumina TruSeq Stranded Total RNA reagents and protocol, except for the control 1 set of CD4+ and CD14+ samples, for which library preparation failed. Reads were mapped using STAR software version 2.7.3a on the GRCh38 genome with GENCODE annotation version 32. Duplicate reads were removed, and reads counted (Supplementary Table 3, https://onlinelibrary.wiley.com/doi/10.1002/art.42396). Final count matrices were analyzed using edgeR software version 3.28.1 to perform normalization and differential expression analysis. Differentially expressed genes were called with an adjusted *P* value of 0.1 (false discovery rate [FDR] of 10%). Functional enrichment analyses were performed with g:Profiler (35) using default settings.

### Linking differential expression and differential interactions in CD4+ T cells versus CD14+ monocytes

Genes corresponding with the promoter end of significant differential interactions observed between CD4+ T cells and CD14+ monocytes were overlapped with those differentially expressed. One‐sided Fisher's exact test was performed to calculate the enrichment of genes with differential interactions in those differentially expressed. In this set of overlapping genes, Spearman's rank correlation was performed to test the correlation of log_2_ fold change values in differential interactions and differential expression. Finally, to test the distribution of log_2_ fold change values, a binomial exact test was performed on a subset of overlapping genes obtained, adding a more stringent cutoff (absolute value of median log_2_ fold change >2 for each gene). Functional enrichment analyses were performed with g:Profiler (35) using default settings.

### Defining SSc‐associated GWAS loci

All independent, non–major histocompatibility complex, disease‐associated signals for SSc were selected from the largest GWAS meta‐analysis performed to date (9). We defined 23 regions based on linkage disequilibrium data and SNP proximity from the total 27 independent signals identified by GWAS. The window ranges and total number of SNPs in each of the 23 final loci are specified in Supplementary Table 4, https://onlinelibrary.wiley.com/doi/10.1002/art.42396.

### Identifying expression QTL genes for SSc‐associated GWAS loci

Publicly available expression QTL data for isolated immune cells were downloaded from the Database of Immune Cell Expression's expression QTL database (36), and blood expression QTL data from the Expression QTLGen Consortium (second release) database (37). Genes were linked to each GWAS locus if the lead SNP was linked to a gene in the respective databases.

### Defining enhancers and transcription activation domains in CD4+ T cells and CD14+ monocytes

To define enhancer regions, ChromHMM version 1.22 annotations from 9 CD4+ T cells and 4 CD14+ monocytes were downloaded from the EpiMAP project (38). For each cell type, enhancer regions were defined as those with a state number from ChromHMM corresponding to enhancer activity present in ≥1 sample. Transcription activation domain definitions for CD4+ T cells and CD14+ monocytes were obtained from a prior study by Javierre et al (22).

### Overlap between promoter capture Hi‐C, SSc‐associated GWAS loci, and enhancer regions

To prioritize certain interactions observed in promoter capture Hi‐C data of particular interest in SSc‐associated GWAS loci, the previously defined SNP set was overlapped with enhancer regions of each cell type (Supplementary Table 4, https://onlinelibrary.wiley.com/doi/10.1002/art.42396). This new SNP set was then overlapped with the promoter interacting regions of significant promoter capture Hi‐C interactions, defining candidate interacting genes as those in which their promoter interacting region overlaps with our significant SSc‐associated SNP set and enhancer regions. GWAS analysis of regulatory and functional information enrichment with linkage disequilibrium (GARFIELD) software version 2 (39) was used to estimate the enrichment of the GWAS SNPs in CD4+ T cell and CD14+ monocyte enhancer regions using a *P* value threshold of 1 × 10^−8^. Functional enrichment analyses were performed for the sets of interacting genes observed in CD4+ T cells and CD14+ monocytes with g:Profiler (35).

### Drug target analysis

To assess if genes interacting with SSc‐associated GWAS loci in CD4+ T cells and CD14+ monocytes were potential drug targets that could be repurposed for use in SSc, those interacting genes with a promoter interacting region overlapping significant SSc‐associated GWAS SNPs and enhancer regions were used to model a protein–protein interaction network using STRING software version 11 (40) (Supplementary Table 5, https://onlinelibrary.wiley.com/doi/10.1002/art.42396). Protein products from these genes and those in direct protein–protein interaction with them were used to query the Open Targets Platform for drug targets (https://platform.opentargets.org). Additionally, the same platform and the DrugBank database (https://www.drugbank.com) were searched for information on clinical studies of drug targets of interest in SSc.

### Complete methods and data availability statement

For more details about this study's methods, please see the Supplementary Material at https://onlinelibrary.wiley.com/doi/10.1002/art.42396. The processed data sets generated from this study are available in the Gene Expression Omnibus repository under the accession number GSE212100. Raw reads and genotypes can be provided upon reasonable request from the authors.

## RESULTS

In this study, we generated promoter capture Hi‐C data for CD4+ T cells and CD14+ monocytes from 10 SSc patients and 5 healthy controls. CHiCAGO software was used to identify significant interactions (CHiCAGO score >5) for each cell type and disease condition (Supplementary Table 6, available at https://onlinelibrary.wiley.com/doi/10.1002/art.42396), and Chicdiff software was used to identify differential interactions between cell types and between disease conditions for each cell type. A total of 81,624 and 74,853 significant interactions originating from 8,193 and 7,024 captured promoters were identified in CD4+ T cells and CD14+ monocytes, respectively. Through integration with published ChIP‐seq data, we found that promoter interacting regions were enriched in H3K27ac and H3K4me3 histone marks from primary CD4+ naive T cells and CD14+ monocytes (Supplementary Figure 2), suggesting that promoters are more likely to interact with active regulatory regions such as enhancers.

### Differential interactions and expression between SSc patients and healthy controls

We first attempted to identify specific interactions that could be present in SSc patients but not in healthy controls, or vice versa, and thus identify specific genes interacting with enhancer regions and SSc‐associated GWAS loci that could be of interest in SSc pathology. We identified a total of 4,858 significant differential interactions (weighted adjusted *P* < 0.05) between SSc patients and healthy controls in CD4+ T cells originating from 1,526 captured promoters, although the significance was modest (median weighted adjusted *P* = 2.2 × 10^−2^) as compared with differential interactions between cell types (median weighted adjusted *P* = 2.16 × 10^−10^). Moreover, we could not detect any significant differential interactions in CD14+ monocytes, indicating weak differences in cells isolated from blood between patients and healthy controls. None of the 23 SSc‐associated GWAS regions showed significant differences at the interaction level between patients and controls.

Regarding transcriptome differences, we identified a total of 62 and 63 differentially expressed genes (FDR <10%) between patients and controls in CD4+ T cells and CD14+ monocytes, respectively (Supplementary Tables 7 and 8, https://onlinelibrary.wiley.com/doi/10.1002/art.42396). In CD4+ T cells we observed significant enrichment in pathways related to immune response, such as positive regulation of immune system process or leukocyte activation (Supplementary Table 9). However, we could not identify any functional enrichment regarding the 63 genes differentially expressed in CD14+ monocytes. Taken together, these results indicate that only modest differences are present in cells isolated from peripheral blood from SSc patients compared to those from healthy controls.

### Identification of allele‐associated chromatin interactions

Recently there have been reports of allele‐associated chromatin interactions; however, these studies were limited in size or had low resolution and were based in cell lines (25, 26). We wanted to test if allele‐associated interactions were present in our data set of primary cells. For each individual, we assigned reads based on the haplotype of origin and quantified allelic imbalance in the read counts for all the CHiCAGO significant loops. We then aggregated the results for all the samples which were heterozygous for that SNP. After stringent quality control, we identified 577 and 541 SNP loop pairs in CD4+ T cells and CD14+ monocytes, respectively (FDR <5%) (Supplementary Data 1 and 2, https://onlinelibrary.wiley.com/doi/10.1002/art.42396), representing 171 and 139 allele‐associated loops in CD4+ T cells and CD14+ monocytes, respectively.

None of the SSc‐associated GWAS SNPs displayed allelic imbalance interactions at this statistical power; however, we still identified allele‐associated interactions with important genes related to immunity. For example, in CD4+ T cells, we found allele‐associated interactions connecting the SNP rs661849 (located downstream of *IRF6*) and *TRAF3IP3* and *IRF6*. Interestingly, this SNP is also an expression QTL for these 2 genes, further validating our approach. In CD14+ monocytes, we identified allele‐associated interactions linking a group of SNPs located around the promoter of *GPX3* and the promoters of *GPX3* and *TNIP1*. Again, these SNPs were also identified as expression QTLs for both genes, although these specific SNPs were not found to be associated with any disease in the GWAS catalog. Overall, we identified allele‐associated interactions between SNPs and 62 and 57 genes in CD4+ T cells and CD14+ monocytes, respectively. Among these interactions, 38 and 31 SNPs in CD4+ T cells and CD14+ monocytes, respectively, were also expression QTLs for those genes in the Expression QTLGen Consortium database (4 and 3 SNPs, respectively, in the Database of Immune Cell Expression's expression QTL database).

### Linking differential expression and differential interactions in CD4+ T cells versus CD14+ monocytes

Next, we decided to look at differences at the interaction and expression level between cell types and how these correlate with each other, without taking disease state into account. We identified 2,257 strongly differentially expressed genes (absolute log_2_ fold change >2; FDR <5%) between CD4+ T cells and CD14+ monocytes, of which 919 and 1,338 genes were overexpressed in CD4+ T cells and CD14+ monocytes, respectively. Overrepresentation analyses showed that each group of genes was, as expected, significantly enriched in T cell–specific and monocyte‐specific pathways, including gene ontology, such as T cell activation and T cell differentiation in CD4+ T cells and leukocyte activation in CD14+ monocytes (Supplementary Tables 10 and 11, https://onlinelibrary.wiley.com/doi/10.1002/art.42396).

Regarding the interactome, we identified 71,213 significant differential interactions (weighted adjusted *P* < 0.05) originating from 8,223 captured promoters. We observed that differentially expressed genes were significantly enriched in differentially interacting genes (Fisher's exact test *P* = 3.54 × 10^−37^, odds ratio [OR] for enrichment 1.77). Furthermore, from the total of 1,209 differentially expressed genes overlapping differentially interacting genes, we observed that genes overexpressed in a specific cell type significantly correlated with increased number of chromatin interactions in that cell type and vice versa (Spearman's rank correlation ρ = 0.73, *P* = 1.04 × 10^−197^).

Finally, we applied a more stringent cutoff in differentially interacting genes (absolute log_2_ fold change >2), leading to a total of 97 differentially expressed genes overlapping differential interactions. In this subset, only 2 of the 97 genes did not behave as expected; 23 and 72 genes were overexpressed and presented an increased number of interactions in CD4+ T cells and CD14+ monocytes, respectively (exact binomial test *P* = 6.01 × 10^−26^; probability of success = 98%) (Supplementary Figure 3, https://onlinelibrary.wiley.com/doi/10.1002/art.42396). Thus, our results showed that chromatin conformation is highly specific to cell type and linked to gene expression, demonstrating the importance of using the correct cell types to define promoter interactions and linking genes to GWAS loci.

### SSc‐associated GWAS loci and CD4+ and CD14+ promoter interactions

Finally, we identified new potential target genes for SSc‐associated GWAS loci, as well as the potential implication of different cell types in gene associations. We performed a multiomic analysis overlapping 23 regions defined by the most powerful SSc‐associated GWAS meta‐analysis performed to date (9) with enhancer regions and our promoter capture Hi‐C data (see Supplementary Material, https://onlinelibrary.wiley.com/doi/10.1002/art.42396). In addition, we also integrated our findings with 2 large expression QTL databases, Expression QTLGen Consortium, which is the largest blood expression QTL meta‐analysis (37), and Database of Immune Cell Expression's expression QTL database, which is the largest study that makes use of purified immune cell populations (36) (Supplementary Data 3, https://onlinelibrary.wiley.com/doi/10.1002/art.42396).

Among 1,505 total SNPs with genome‐wide significance (*P* < 5 × 10^−8^) and SNPs associated with SSc and those in high linkage disequilibrium (r^2^ > 0.8), 445 (29.6%) and 284 (18.9%) SNPs overlapped with enhancer regions from CD4+ T cells and CD14+ monocytes, respectively. As expected, the GWAS SNPs significantly enriched enhancer regions in both CD4+ T cells (OR for enrichment by GARFIELD test 3.40, *P* = 6.7 × 10^−4^) and CD14+ monocytes (OR for enrichment by GARFIELD test 3.05, *P* = 1.7 × 10^−3^). In addition, the differences in the number of SNPs overlapping CD4+ and CD14+ enhancer regions were significant (2‐proportion Z test *P* = 0.001), showing a stronger enrichment with CD4+ T cell enhancer regions compared with that observed in CD14+ monocytes. These GWAS SNPs within enhancer regions were overlapped with promoter interacting regions from promoter capture Hi‐C, resulting in a total 398 and 109 significant interactions in CD4+ T cells and CD14+ monocytes, respectively (Supplementary Table 4). The promoter ends of those interactions corresponded to 46 genes, with a total of 40 and 27 interacting genes in CD4+ T cells and CD14+ monocytes, respectively (Table 1).

**Table 1:** Promoter capture Hi‐C target genes for the 23 SSc‐associated regions in CD4+ T cells and CD14+ monocytes*

Chr	Base position (start–end)†	GWAS locus‡	Promoter capture Hi‐C target genes§		CD4+ versus CD14+	
			CD4+ T cells	CD14+ monocytes	Differential interactions	Differential expression
1	67326053–67448804	rs3790566 *(IL12RB2)*	–	–	–	–
1	167445635–167465040	rs2056626 *(CD247)*	***CD247**, CREG1*	–	***CD247**, CREG1*	***CD247**, CREG1*
1	173337507–173391947	rs1857066 *(TNFSF4‐LOC100506023‐PRDX6)*	–	–	–	–
2	190642047–190698201	rs16832798 *(NAB1)*	*MFSD6, NEMP2*	*MFSD6, NEMP2, HIBCH;INPP1*	*MFSD6, NEMP2, HIBCH;INPP1*	*MFSD6, NEMP2, HIBCH, INPP1*
2	191035723–191108308	rs3821236 *(STAT4)*	***STAT4**, NABP1*	–	***STAT4**, NABP1*	***STAT4**, NABP1*
3	58084620–58482701	rs4076852 *(FLNB‐DNASE1L3‐PXK)*	*RPP14, KCTD6*	*RPP14, KCTD6*	*RPP14, KCTD6*	*KCTD6*
3	119384733–119546340	rs9884090 *(POGLUT1‐TIMMDC1‐CD80‐ARHGAP31)*	*TMEM39A;POGLUT1*	–	*TMEM39A;POGLUT1*	*TMEM39A, POGLUT1*
3	160002484–160030580	rs589446 *(IL12A)*	–	*SMC4;IFT80*	*SMC4;IFT80*	*SMC4, IFT80*
4	960523–990021	rs11724804 *(DGKQ)*	*GAK;TMEM175, FGFRL1*	*GAK;TMEM175, FGFRL1*	*FGFRL1*	*GAK, TMEM175, FGFRL1*
4	102477892–102615256	rs230534 *(NFKB1)*	*SLC39A8, **NFKB1**, UBE2D3;CISD2, SLC9B1, BDH2*	*SLC39A8, UBE2D3;CISD2, BDH2*	*SLC39A8, **NFKB1**, UBE2D3;CISD2, SLC9B1, BDH2*	*SLC39A8, UBE2D3, CISD2, BDH2*
5	151064651–151080486	rs3792783 *(TNIP1)*	–	–	–	–
6	106181815–106339294	rs633724 *(ATG5)*	–	–	–	–
7	128933913–129095960	rs36073657 *(IRF5‐TNPO3)*	–	–	–	–
8	11474517–11544554	rs2736340 *(FAM167A‐BLK)*	–	–	–	–
8	60638547–60664239	rs685985 *(RAB2A‐CHD7)*	*ASPH, SDCBP, CHD7*	–	*ASPH, SDCBP*	*ASPH, SDCBP, CHD7*
11	554659–619789	rs6598008 *(CDHR5‐IRF7)*	–	–	–	–
11	2311894–2363262	rs2651804 *(TSPAN32,CD81‐AS1)*	*TSSC4*	–	*TSSC4*	*TSSC4*
11	118704617–118875175	rs11217020 *(DDX6)*	*CXCR5, UPK2, **DDX6**, IFT46;ARCN1*	*CXCR5*	*CXCR5, UPK2, **DDX6***	*CXCR5, **DDX6,** ARCN1*
15	74739180–75148328	rs1378942 *(CSK)*	***CSK**, CLK3, ULK3,SCAMP2, MPI, FAM219B, COX5A*	***CSK**, CLK3, ULK3, SCAMP2, MPI, FAM219B, COX5A, C15orf39*	***CSK**, ULK3, SCAMP2, MPI, FAM219B, COX5A, C15orf39*	***CSK**, CLK3, ULK3, SCAMP2, MPI, FAM219B, COX5A, C15orf39*
16	85932852–85979945	rs11117420 *(IRF8)*	–	***IRF8***	***IRF8***	***IRF8***
17	39747478–39933464	rs883770 *(IKZF3‐GSDMB)*	***IKZF3**, ERBB2, PSMD3*	*ERBB2*	***IKZF3**, ERBB2, PSMD3*	***IKZF3**, ERBB2, PSMD3*
17	75193533–75279345	rs1005714 *(NUP85‐GRB2)*	–	–	–	–
19	18068862–18093031	rs2305743 *(IL12RB1)*	*PIK3R2, RAB3A*	*RAB3A*	*PIK3R2, RAB3A*	–

* Genes classically associated with systemic sclerosis (SSc) through proximity to genome‐wide association study (GWAS) loci are set in bold. Chr = chromosome.

† Base position in GRCh38 (hg 38) assembly.

‡ Locus as defined by López‐Isac et al (ref. 9).

§ Genes corresponding with promoter interacting regions overlapping enhancer regions and SSc‐associated GWAS single‐nucleotide polymorphisms.

**Figure 1:**
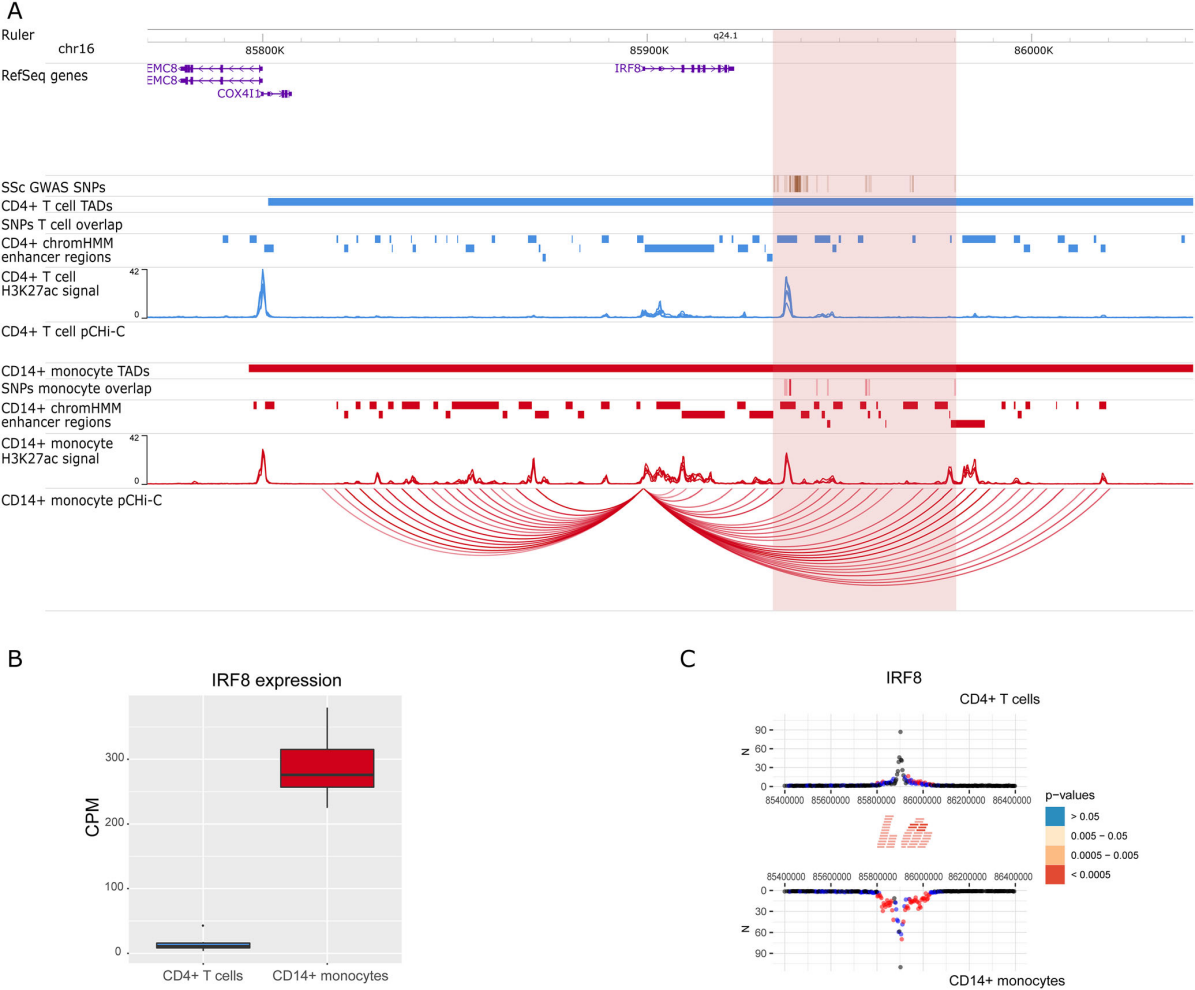
Promoter capture Hi‐C (pCHi‐C) interactions and gene expression in the rs11117420 (*IRF8*) genome‐wide association study (GWAS) locus. **A**, Genomic coordinates (GRCh38) are shown at the top of the panel. The tracks include NCBI RefSeq genes, systemic sclerosis (SSc)–associated GWAS single‐nucleotide polymorphisms (SNPs) from López‐Isac et al (ref. 9) and those in high linkage disequilibrium (LD) (r^2^ > 0.8), transcription activation domains (TADs) (shown as bars), SNPs overlapping promoter interacting regions and enhancer regions, enhancer regions as defined by ChromHMM software, H3K27ac signal, and pCHi‐C significant interactions (CHiCAGO score >5) (shown as arcs) in CD4+ T cells (blue) and CD14+ monocytes (red). The red highlighted region includes the block of all the SSc‐associated SNPs in LD. **B**, Box plot of *IRF8* expression level in CD4+ T cells and CD14+ monocytes in count per million (CPM). Each box represents the 25th to 75th percentiles. Lines inside the boxes represent the median. Lines outside the boxes represent the 10th and 90th percentiles. The dot represents an outlier. **C**, Chicdiff software bait profiles for *IRF8*. The plot shows the raw read counts versus linear distance from the bait fragment as mirror images for CD4+ T cells (top) and CD14+ monocytes (bottom). Other‐end interacting fragments are pooled and color‐coded by their weighted adjusted *P* value. Significant differentially interacting regions detected by Chicdiff overlapping SSc‐associated GWAS SNPs and enhancer regions are depicted as red blocks. Chr = chromosome.

**Figure 2:**
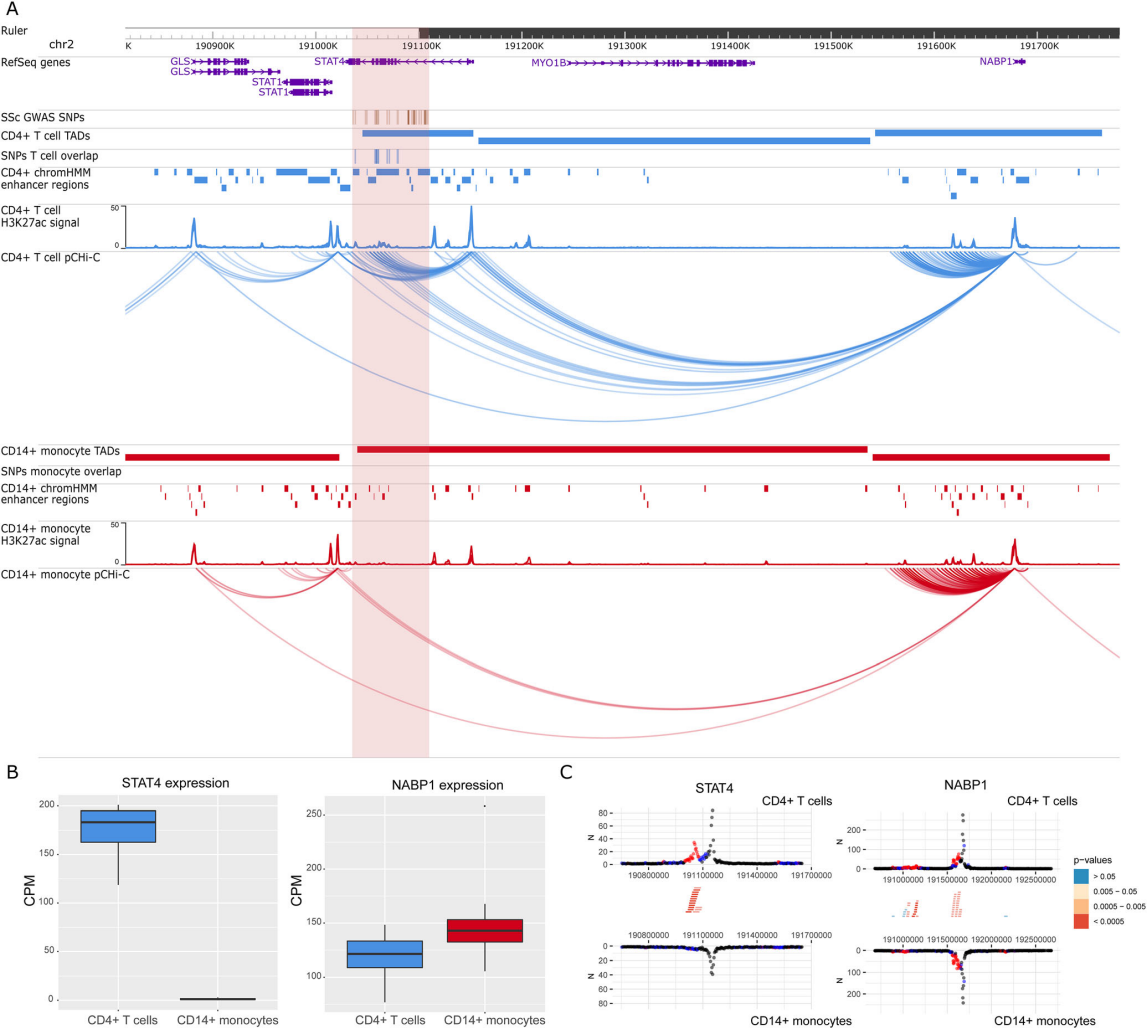
Promoter capture Hi‐C (pCHi‐C) interactions and gene expression in the rs11117420 (*STAT4*) GWAS locus. **A**, Genomic coordinates (GRCh38) are shown at the top of the panel. The tracks include NCBI RefSeq genes, SSc‐associated GWAS SNPs from López‐Isac et al (ref. 9) and those in high LD (r^2^ > 0.8), TADs (shown as bars), SNPs overlapping promoter interacting regions and enhancer regions, enhancer regions as defined by ChromHMM, H3K27ac signal, and pCHi‐C significant interactions (CHiCAGO score >5) (shown as arcs) in CD4+ T cells (blue) and CD14+ monocytes (red). The red highlighted region includes the block of all the SSc‐associated SNPs in LD. **B**, Box plots of *STAT4* (left) and *NABP1* (right) expression levels in CD4+ T cells and CD14+ monocytes in count per million (CPM). Each box represents the 25th to 75th percentiles. Lines inside the boxes represent the median. Lines outside the boxes represent the 10th and 90th percentiles. The dot represents an outlier. **C**, Chicdiff bait profiles for *STAT4* (left) and *NABP1* (right). Plots show the raw read counts versus linear distance from the bait fragment as mirror images for CD4+ T cells (top) and CD14+ monocytes (bottom). Other‐end interacting fragments are pooled and color‐coded by their weighted adjusted *P* value. Significant differentially interacting regions detected by Chicdiff overlapping SSc‐associated GWAS SNPs and enhancer regions are depicted as red blocks. See Figure 1 for other definitions.

**Figure 3:**
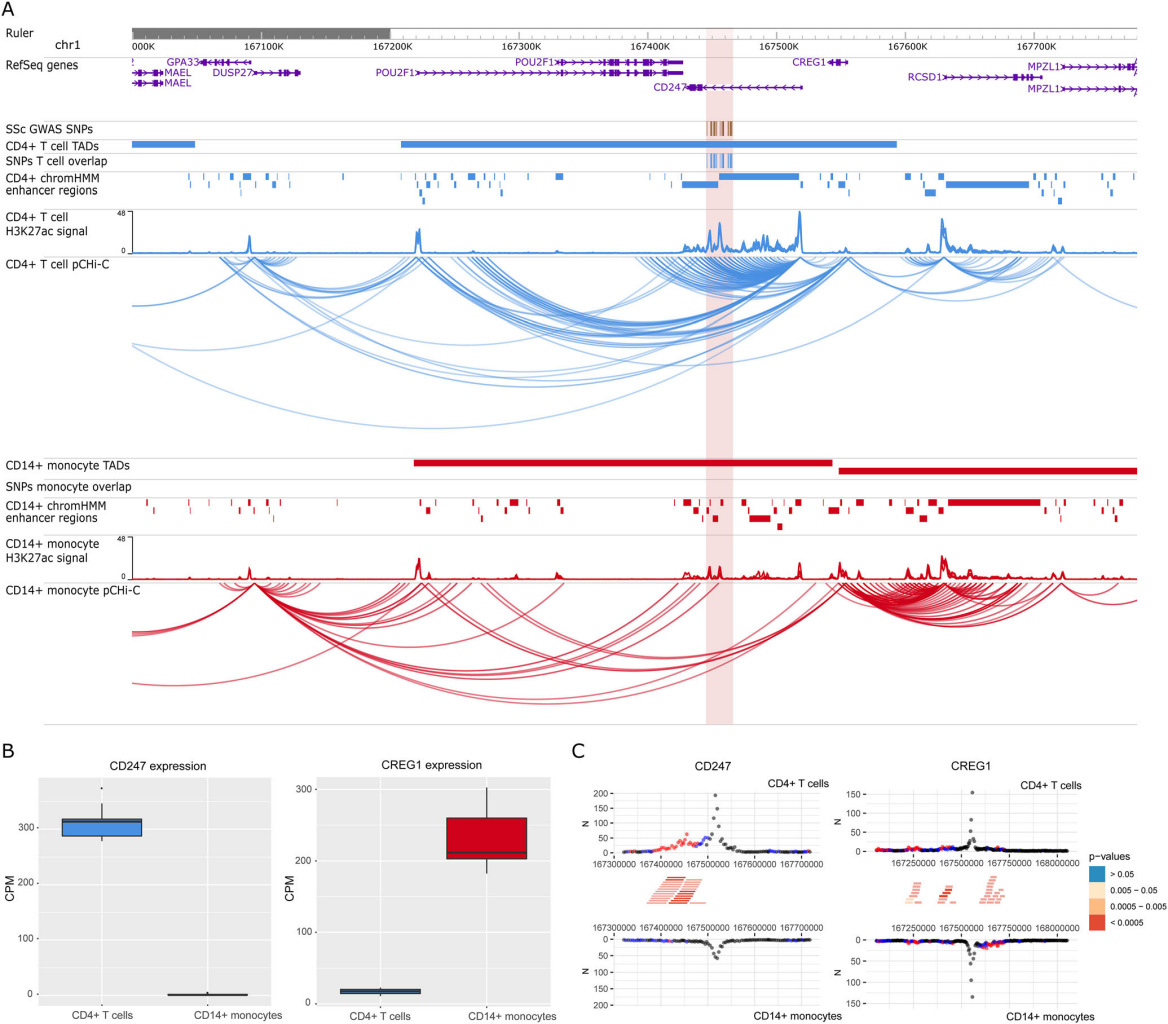
Promoter capture Hi‐C (pCHi‐C) interactions and gene expression in the rs2056626 (*CD247*) GWAS locus. **A**, Genomic coordinates (GRCh38) are shown at the top of the panel. The tracks include NCBI RefSeq genes, SSc‐associated GWAS SNPs from López‐Isac et al (ref. 9) and those in high LD (r^2^ > 0.8), TADs (shown as bars), SNPs overlapping promoter interacting regions and enhancer regions, H3K27ac signal, enhancer regions as defined by ChromHMM, and pCHi‐C significant interactions (CHiCAGO score >5) (shown as arcs) in CD4+ T cells (blue) and CD14+ monocytes (red). The red highlighted region includes the block of all the SSc‐associated SNPs in LD. **B**, Box plots of *CD247* (left) and *CREG1* (right) expression levels in CD4+ T cells and CD14+ monocytes in count per million (CPM). Each box represents the 25th to 75th percentiles. Lines inside the boxes represent the median. Lines outside the boxes represent the 10th and 90th percentiles. The dot represents an outlier. **C**, Chicdiff bait profiles for *CD247* (left) and *CREG1* (right). Plots show the raw read counts versus linear distance from the bait fragment as mirror images for CD4+ T cells (top) and CD14+ monocytes (bottom). Other‐end interacting fragments are pooled and color‐coded by their weighted adjusted *P* value. Significant differentially interacting regions detected by Chicdiff overlapping SSc‐associated GWAS SNPs and enhancer regions are depicted as red blocks. See Figure 1 for other definitions.

The interaction maps presented in this study identified 39 new potential candidate genes and confirmed 7 genes that have been previously associated with SSc using genomic proximity. Differential expression and differential interaction data for each of the 46 genes and baited promoters are available in Supplementary Tables 12 and 13, respectively, at https://onlinelibrary.wiley.com/doi/10.1002/art.42396. Interestingly, some genes with confirmed SSc association, such as *IRF8, STAT4*, or *CD247*, showed cell type–specific interactions (Figures 1, 2, 3).

The rs11117420 (*IRF8*) locus (Figure 1) provides a good example in which interactions between SNPs overlapping enhancer regions (represented by H3K27ac mark peaks) and the *IRF8* promoter were found exclusively in 1 cell type, and was associated with differential gene expression between cells, in which CD14+ monocytes showed a much higher expression of *IRF8* (log_2_ fold change = –4.47; FDR = 3.11 × 10^−72^). For the rs3821236 (*STAT4*) locus (Figure 2), significant interactions with the *STAT4* promoter were identified exclusively in CD4+ T cells, corresponding with a transcription activation domain specific for CD4+ T cells that is not found in monocytes. In addition, *STAT4* showed a significantly higher expression in CD4+ T cells compared with CD14+ monocytes (log_2_ fold change = 7.05; FDR = 1 × 10^−304^). Interestingly, neither of these 2 loci showed expression QTL signals to these genes in either database. The rs11117420 SNP was expression QTL for a long noncoding RNA gene (*RP11‐542M13.3*) while rs3821236 only showed weak expression QTL signals for *GLS* and *MFSD6*. Cell type–specific interactions were also observed in the rs2056626 (*CD247*) GWAS locus (Figure 3), in which significant interactions between SNPs and the *CD247* promoter were identified only in CD4+ T cells, with an increased expression of this gene in CD4+ T cells compared with CD14+ monocytes (log_2_ fold change = 7.49; FDR = 3.99 × 10^−210^). We also found that rs2056626 was a strong expression QTL for *CD247*.

**Figure 4:**
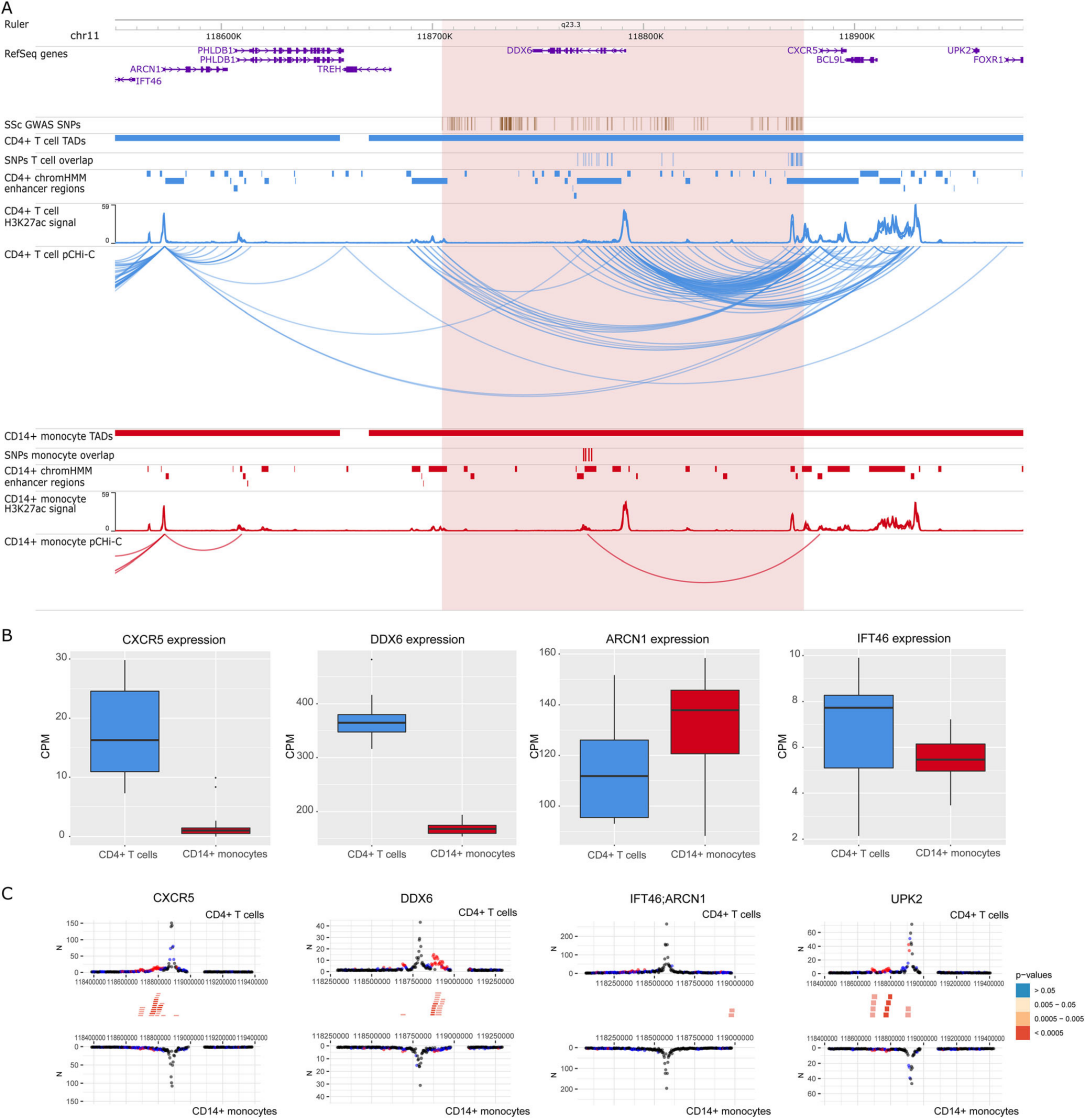
Promoter capture Hi‐C (pCHi‐C) interactions and gene expression in the rs11217020 (*DDX6*) GWAS locus. **A**, Genomic coordinates (GRCh38) are shown at the top of the panel. The tracks include NCBI RefSeq genes, SSc‐associated GWAS SNPs from López‐Isac et al (ref. 9) and those in high LD (r^2^ > 0.8), TADs (shown as bars), SNPs overlapping promoter interacting regions and enhancer regions, enhancer regions as defined by ChromHMM, H3K27ac signal, and pCHi‐C significant interactions (CHiCAGO score >5) (shown as arcs) in CD4+ T cells (blue) and CD14+ monocytes (red). The red highlighted region includes the block of all the SSc‐associated SNPs in LD. **B**, Box plots of *CXCR5*, *DDX6*, *ARCN1*, and *IFT46* expression levels in CD4+ T cells and CD14+ monocytes in count per million (CPM). Each box represents the 25th to 75th percentiles. Lines inside the boxes represent the median. Lines outside the boxes represent the 10th and 90th percentiles. Dots represents outliers. **C**, Chicdiff bait profiles for *CXCR5*, *DDX6*, *IFT46/ARCN1* (shared capture bait), and *UPK2*. Plots show the raw read counts versus linear distance from the bait fragment as mirror images for CD4+ T cells (top) and CD14+ monocytes (bottom). Other‐end interacting fragments are pooled and color‐coded by their weighted adjusted *P* value. Significant differentially interacting regions detected by Chicdiff overlapping SSc‐associated GWAS SNPs and enhancer regions are depicted as red blocks. See Figure 1 for other definitions.

In addition, we identified new potential candidate genes interacting with SSc‐associated GWAS SNPs. For example, in the rs11217020 (*DDX6*) locus (Figure 4), we found significant interactions between SNPs overlapping enhancer regions, and not only *DDX6* but also other potential candidate genes, including *CXCR5*, *UPK2*, and *IFT46/ARCN1* promoters in CD4+ T cells. In CD14+ monocytes, only a significant interaction with *CXCR5* promoter was found. All of these interactions are within their own transcription activation domain, except for the interaction including *IFT46/ARCN1* promoters, and we observed a significantly higher gene expression of *CXCR5* (log_2_ fold change = 3.21; FDR = 1.05 × 10^−09^) and *DDX6* (log_2_ fold change = 1.14; FDR = 2.38 × 10^−83^) in CD4+ T cells, while *ARCN1* showed a slight overexpression in CD14+ monocytes (log_2_ fold change = –0.21; FDR = 3.69 × 10^−03^). *ARCN1* and *CXCR5* were also expression QTL hits for this SNP in blood (Supplementary Data 3), further supporting their role in SSc pathogenesis.

To identify what pathways could be driving disease in the 2 different cell types, we performed a functional enrichment analysis including the genes interacting with SSc‐associated GWAS loci for each cell type. In CD4+ T cells, the set of 40 interacting genes observed showed enrichment in virus response and pancreatic carcinoma (Supplementary Table 14, https://onlinelibrary.wiley.com/doi/10.1002/art.42396). In accordance with this, a higher incidence of cancer in SSc patients compared with the general population has been suggested in several studies (41). On the other hand, the set of 27 interacting genes observed in CD14+ monocytes showed enrichment in tyrosine kinase activity (Supplementary Table 15), which plays an important role in fibrosis and has been related with SSc pathogenesis, making tyrosine kinase inhibitors one of the most promising antifibrotic therapies for SSc and other fibrotic diseases (42).

Plots of the interactions for the rest of the SSc‐associated GWAS loci in CD4+ T cells and CD14+ monocytes can be found in Supplementary Figures 4–22. All expression QTL hits are available in Supplementary Data 3, available at https://onlinelibrary.wiley.com/doi/10.1002/art.42396.

### Potential targets for drug repurposing

As a proof of concept, we wanted to determine the potential for these genes as novel treatment options for SSc. From the 46 genes that presented promoter interacting regions overlapping significant SSc‐associated GWAS SNPs and enhancer regions, we identified a total of 21 drugs of interest in SSc that target protein products that are in strong protein–protein interaction with 13 of those genes (5 of them specific for CD4+ T cells interactions) (Table 2). Fifteen of these drugs are potential drug targets already in use, or have at least completed clinical phase III trials, in other similar immune‐mediated diseases that could be repurposed for SSc treatment, such as metformin or dimethyl fumarate. Tocilizumab and nintedanib were 2 of the potential drugs highlighted in our analysis, both of them approved by the Food and Drug Administration for use in SSc‐associated interstitial lung disease (43, 44). We also identified 4 drugs currently in advanced clinical trials in SSc (tofacitinib, bosentan, methylprednisolone, and mycophenolic acid).

**Table 2:** Summary of potential targets for drug repurposing in SSc based on promoter capture Hi‐C data

Genome‐wide association study locus	Promoter capture Hi‐C interacting genes	Cell type with interactions	Genes in strong protein–protein interaction	Targeted drug	Disease indication*
rs2056626	*CREG1*	CD4+ T cells	*TUBB4B*	Colchicine	Osteoarthritis, advanced fibrosis
rs4076852	*RPP14*	CD4+ T cells, CD14+ monocytes	*KEAP1*	Dimethyl fumarate	Psoriasis, multiple sclerosis, disseminated sclerosis
			*AGTR1*	Candesartan	Type 1 diabetes mellitus
			*HSPA8*	Forigerimon	Systemic lupus erythematosus
rs230534	*NFKB1*	CD4+ T cells	*IL12B*	Ustekinumab	Psoriasis, Crohn's disease, ulcerative colitis
			*IL1R1*	Anakinra	Rheumatoid arthritis
			*IL23A*	Tildrakizumab	Psoriasis
			*JAK2*	Tofacitinib	**SSc**, rheumatoid arthritis, ulcerative colitis, interstitial lung disease, Takayasu arteritis
			*NR3C1*	Methylprednisolone†	Rheumatoid arthritis, Crohn's disease, psoriatic arthritis, ulcerative colitis, Behçet's syndrome
	*UBE2D3*	CD4+ T cells, CD14+ monocytes	*KEAP1*	Dimethyl fumarate	Psoriasis, multiple sclerosis, disseminated sclerosis
rs685985	*SDCBP*	CD4+ T cells	*IMPDH1*	Mycophenolic acid†	Systemic lupus erythematosus, immunosuppression
			*TUBB4B*	Colchicine	Osteoarthritis, advanced fibrosis
	*CHD7*	CD4+ T cells	*PPARG*	Mesalamine	Crohn's disease, ulcerative colitis
rs11217020	*CXCR5*	CD4+ T cells, CD14+ monocytes	*S1PR3*	Fingolimod	Multiple sclerosis, disseminated sclerosis
rs1378942	*CSK*	CD4+ T cells, CD14+ monocytes	*FLT4*	Nintedanib	**SSc**, idiopathic pulmonary fibrosis, interstitial lung disease
	*COX5A*	CD4+ T cells, CD14+ monocytes	*NDUFB10*	Metformin	Type 1 diabetes mellitus, type 2 diabetes mellitus
rs883770	*IKZF3*	CD4+ T cells	*JAK1*	Baricitinib	Rheumatoid arthritis
			*JAK3*	Upadacitinib	Rheumatoid arthritis
			*IL2RA*	Basiliximab	Type 1 diabetes mellitus
	*ERBB2*	CD4+ T cells, CD14+ monocytes	*IL6R*	Tocilizumab	**SSc**, rheumatoid arthritis, juvenile idiopathic arthritis, giant cell arteritis
			*JAK* kinases	Tofacitinib	**SSc**, rheumatoid arthritis, ulcerative colitis, interstitial lung disease, Takayasu arteritis
rs2305743	*PIK3R2*	CD4+ T cells	*ADRA1B*	Epinephrine	Crohn's disease
			*AGTR1*	Candesartan	Type 1 diabetes mellitus
			*EDNRA*	Bosentan	**SSc**, idiopathic pulmonary fibrosis, pulmonary arteria hypertension
			*JAK1*	Baricitinib	Rheumatoid arthritis
			*JAK* kinases	Tofacitinib	**SSc**, rheumatoid arthritis, ulcerative colitis, interstitial lung disease, Takayasu arteritis
			*PDGFRB*	Nintedanib	**SSc**, idiopathic pulmonary fibrosis, interstitial lung disease
	*RAB3A*	CD4+ T cells, CD14+ monocytes	*HSPA8*	Forigerimod	Systemic lupus erythematosus

* Only related immune‐mediated diseases listed. All clinical trials for use in these diseases at least completed phase III.

† Drugs currently in phase III or earlier phase clinical trials in systemic sclerosis (SSc).

## DISCUSSION

Our study of SSc genetics integrated GWAS, chromosome conformation, gene expression, and cell specificity. Our findings stress the importance of using the correct cell type in the functional interpretation of GWAS associations. We identified new target genes and confirmed others in SSc‐associated GWAS loci in 2 of the main cell types associated with the disease, CD4+ T cells and CD14+ monocytes. We also further validated the presence of interactions that are altered by common genomic variations and showed how these are correlated with expression QTLs.

One of the new candidate genes observed in our promoter capture Hi‐C data corresponded with *CXCR5* in the *DDX6* GWAS locus (Figure 4). *CXCR5* has an important role in the differentiation of follicular helper T cells, and is highly expressed in CD4+ and CD8+ T cells (45). In addition, a recent study observed that follicular helper T cells (CD4+CXCR5+PD‐1+) were increased in SSc and correlated with SSc severity (46). In line with the above, interactions with the promoter of this gene were identified specifically in CD4+ T cells in our study, and the gene's expression was specific to this cell type. Furthermore, *CXCR5* has been associated with other similar immune‐mediated diseases through GWAS studies, such as rheumatoid arthritis and inflammatory bowel disease (47, 48). Thus, *CXCR5* could be a candidate gene contributing to SSc pathology, particularly in CD4+ T cells.

Another interesting finding was the rs685985 (*RAB2A‐CHD7*) locus, a recently discovered locus associated with SSc (9) (Supplementary Figure 16, https://onlinelibrary.wiley.com/doi/10.1002/art.42396). Within this region, we observed significant interactions between SSc‐associated GWAS SNPs and the closest gene, *CHD7*, in CD4+ T cells. *CHD7* is a chromatin remodeler that has been associated with counts of lymphocytes and other immune‐related cells in blood through GWAS (49). Regarding the rs589446 (*IL12A*) locus (Supplementary Figure 9), we identified long‐range interactions between SSc‐associated GWAS SNPs and the promoter of *SMC4* in CD14+ monocytes. *SMC* family genes play a central role in organizing and compacting chromosomes. A recent study showed that *SMC4* promotes an inflammatory innate immune response, which is directly associated with monocyte activity, through enhancing nuclear factor κB essential modulator transcription, an essential modulator of NF‐κB (50). Although *IL12A* has been traditionally considered the most probable candidate gene for this association, we did not observe any interactions between SSc‐associated GWAS SNPs and the promoter of this gene.

Here, it is important to note the increased difficulty to identify significant short‐range interactions (<50 kb), as background read count levels are dependent on the distance between fragments (29). This phenomenon represents a limitation in this kind of study, as most of the GWAS SNPs are classically related with the closest gene and, in some cases, these SNPs are located within the gene itself. In this regard, newer high‐resolution Hi‐C methods should help overcome this limitation of detecting very short‐range interactions (51).

Regarding previously confirmed genes associated with SSc, we observed interactions between *IRF8* promoter and SSc‐associated GWAS SNPs that were only present in CD14+ monocytes (Figure 1), corresponding with an up‐regulated expression of this gene in CD14+ monocytes compared with CD4+ T cells. This transcription factor plays an important role in differentiation and regulation of monocytes and macrophages (52). Furthermore, variants in *IRF8* have been associated with monocyte counts across different populations (53), and down‐regulation of *IRF8* in monocytes and macrophages of SSc patients that may affect the fibrotic phenotype of the disease have been reported (54). A recent study in a mouse model demonstrated that the deletion of an enhancer region corresponding with our SSc‐associated GWAS locus decreased *Irf8* expression, resulting in overproduction of inflammatory Ly6c+ monocytes (55). Thus, our results confirm the association of *IRF8* with SSc through physical chromatin interactions, particularly in CD14+ monocytes. Given the evidence, this locus seems very likely to affect *IRF8*; however, we did not find evidence of expression QTL signals in the 2 databases we explored. This is likely due to limitations of expression QTL studies, which require very large data sets to identify signals and are very laborious to apply to all possible cell populations (in this case monocytes). A recent study also identified limitations in the design of expression QTL studies when used to assign genes to GWAS loci (56).

Other loci confirmed by our study, *CD247* and *STAT4*, have been described in many previous GWAS studies as main candidate genes associated with SSc (9, 10). In our study, interactions were exclusively found in CD4+ T cells (Figures 2, 3). These findings are in line with the literature, as both genes play an important role, particularly in T cell signaling and differentiation (57, 58). Thus, our results highlight the importance of studying GWAS signals with the specific cell types in which interactions are found, acting as a starting point for follow‐up functional studies that can relate these signals with the disease.

Our results revealed that 3‐dimensional chromatin structure is largely preserved between SSc patients and healthy controls, at least in CD4+ T cells and CD14+ monocytes derived from peripheral blood. So far, there is only 1 published study that attempted to observe differences at the interaction level between patients and healthy controls in CD4+ T cells from juvenile idiopathic arthritis patients (59). However, no differences at the interactome level were observed, which supports our hypothesis and emphasizes the difficulty to describe these subtle differences with current technology. Interestingly, it has been shown that subtle differences in chromatin interactions may be correlated with large functional effects on gene expression (25). More significant differences could be expected if cells were isolated from the site of active disease, and further studies involving these samples would be of great interest. On the other hand, we identified many significant allele‐associated interactions, and our study is the first to show that this analysis is possible in primary cells isolated from patients. We found that many of the SNPs associated with these loops are also expression QTLs for the genes they interact with. The overall number of loops presenting allelic imbalance was still very low compared to the loops tested (0.2%) but was in line with previous studies (25, 26). We think this is due to difficulties in assigning reads to a specific allele (only reads overlapping phased heterozygous sites can be tested) and the limited number of read pairs in long‐range interactions, consequently limiting statistical power.

Finally, we wanted to describe general differences between cell types at the interaction and expression level and how these are correlated. We observed that overexpressed genes in a specific cell type correlated with an increased number of interactions, and that those genes were enriched in specific pathways related with T cell and monocyte signaling, activation, and differentiation. These results demonstrate that interactions are directly related with the expression of important genes implicated in cell type–specific pathways. Indeed, a recent study observed that disease‐associated genes tend to be connected by cell type–specific interactions (60). Thus, our data presented here will aid future studies in identifying cell types enriched with interactions overlapping GWAS loci.

## AUTHOR CONTRIBUTIONS

All authors were involved in drafting the article or revising it critically for important intellectual content, and all authors approved the final version to be published. Dr. González‐Serna had full access to all of the data in the study and takes responsibility for the integrity of the data and the accuracy of the data analysis.

### Study conception and design

González‐Serna, Shi, Ding, Martin, Orozco.

### Acquisition of data

González‐Serna, Shi, Hankinson, Ding, McGovern, Villanueva‐Martin, Ortego‐Centeno, Callejas, Martin, Orozco.

### Analysis and interpretation of data

González‐Serna, Shi, Kerick, Tutino, Martin, Orozco.

## Supporting information


Disclosure Form

**Appendix S1:** Supplementary Information

**Data S1:** Supplementary file

**Data S2: S**upplementary file

**Data S3:** Supplementary file
